# Perirenal hematoma and delayed contrast metabolism after cerebral intravascular therapy: A case report

**DOI:** 10.1097/MD.0000000000030807

**Published:** 2022-09-30

**Authors:** Yang Li, Xin Zhao, Ying Zhang, Qian Yang, Guoxing Liu, Tao Liu, Xuekai Zhang, Ming Zhou

**Affiliations:** a Shandong University of Traditional Chinese Medicine, Jinan, China; b Department of Encephalopathy, Traditional Chinese Medicine Hospital of Weifang, Weifang, China; c US Center for Chinese Medicine by Beijing University of Chinese Medicine (BUCM), Rockville, MD.

**Keywords:** cerebral angiograph, complication, delayed contrast metabolism, intravascular therapy, perirenal hematoma

## Abstract

Hematomas after percutaneous angiography often occur in the thigh, retroperitoneal, intraperitoneal, or abdominal wall. Renal hematoma after percutaneous angiography is very rare.

**Diagnoses:**

Herein, we present a case of perirenal hematoma and delayed contrast metabolism after cerebral angiograph, which may be caused by improper operation.

**Interventions:**

Conservative treatments which development by multi-disciplinary collaboration.

**Outcomes:**

After treatment, the clinical symptoms of the patients gradually disappeared and the imaging results became negative.

**Conclusion:**

Though the patient missed timely diagnosis and treatment, fortunately no catastrophic events occurred. Meanwhile, the potential causes, diagnosis, and therapeutic management were all discussed.

## 1. Introduction

The renal hematoma, commonly due to iatrogenic injury, is rare but life-threatening complication after endovascular procedures. Careful guide-wire passage under full visual inspection is important to avoid inadvertent vascular injury during percutaneous angiography. Meanwhile close surveillance and the awareness of this rare complication are crucial for the survival. Here, we report a case of perirenal hematoma and delayed contrast metabolism after cerebral intravascular therapy.

## 2. Case report

A 78-year-old man with history of hypertension, diabetes, and cerebral infarction was admitted to our hospital due to dizziness for 2 days. Physical examination was normal. Magnetic resonance angiography of the brain indicated multiple cerebral arteries stenosis. Then, the patient underwent digital silhouette angiography under local anesthesia for the further assessment with iodixanol (iodine concentration 270 mgi/mL; osmotic pressure 290 mOsm/kg H_2_O; Yangtze River Pharm, Jiangsu Province, China). Digital silhouette angiography was performed with femoral catheterization using the Seldinger technique, and angiography showed that the right vertebral artery was severely stenosed (about 90%) and moderate stenosis in the left internal carotid artery (40%). The right vertebral artery stenting was accepted as a reasonable treatment strategy after the consent of the patient. The 6F guide catheter was placed in the right subclavian artery, followed by the supporting microwire along the subclavian artery to reach the brachial artery, and then the Synchro2 microwire was passed through the initial stenosis of the vertebral artery. The 4.0/12 mm ball expansion stent was introduced into the stent along the microwire and covering both ends of the stenosis. The femoral artery puncture point was sutured with a suture device, and the whole procedure was uneventful.

**Figure 1.**
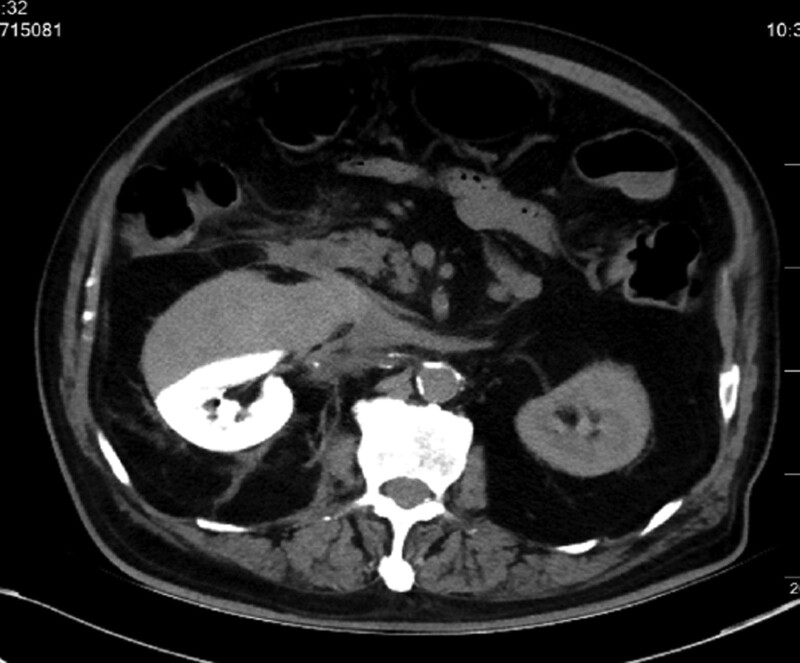
The abdominal computer tomography right revealed perirenal hematoma.

**Figure 2.**
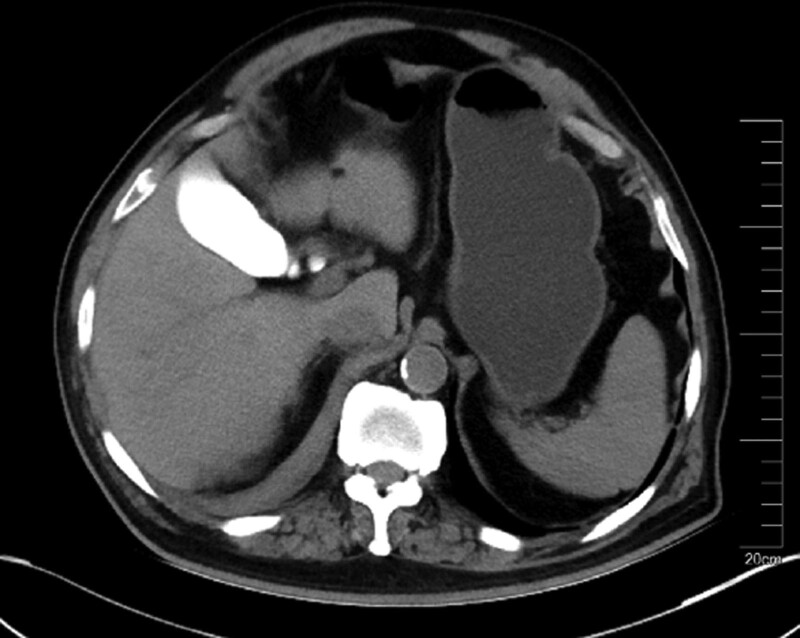
The contrast retention in the right kidney and gallbladder.

However, about 3 hours later he complained of right flank pain, and the physical examination revealed tenderness in right lower abdominal and weakened auscultation bowel sound. Intestinal obstruction was suspected, but there was no relief after enema. His systolic blood pressure was around 120 mm Hg and his vital signs were stable, though the hemoglobin value dropped from 140 to 89 g/L within only 3 hours postoperatively. The following abdominal computer tomography (CT) 1 day later revealed right perirenal hematoma, and contrast retention in the gallbladder and right kidney (Figs. 1 and 2). Renal hemorrhage is considered to be, and the patient was given conservative treatment. It is developed by a multidisciplinary collaboration (Neurology, Interventional surgery, Urology, Nephrology and Imaging) including hydration, diuresis, anti-infection, combination of painkillers, and monitoring of kidney function. His hemodynamics is stable and his kidney function is normal. With the risk of renal hemorrhage and intracranial vascular thrombosis, aspirin monotherapy was given. After treatment, the pain and discomfort were relieved slightly.

**Figure 3.**
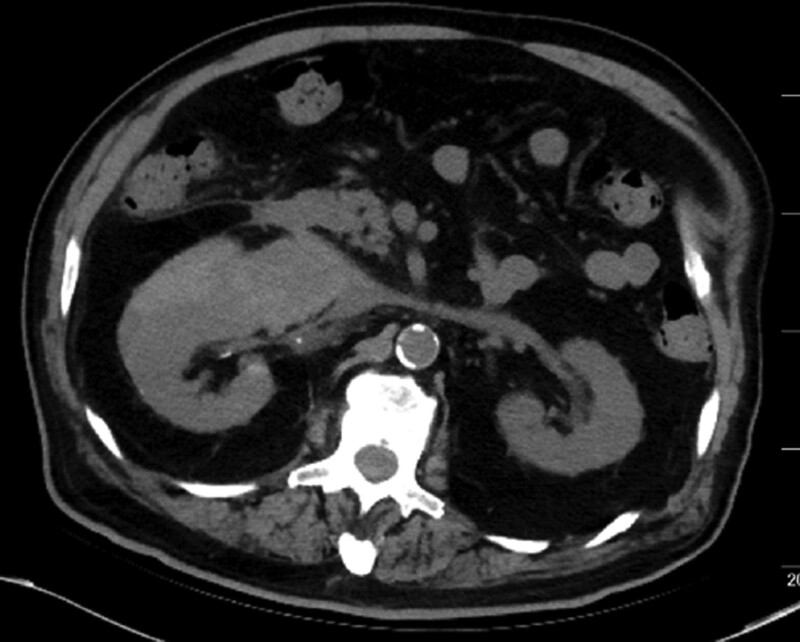
The abdominal computer tomography right revealed the changes of hematoma and peripheral hematocele were not obvious.

**Figure 4.**
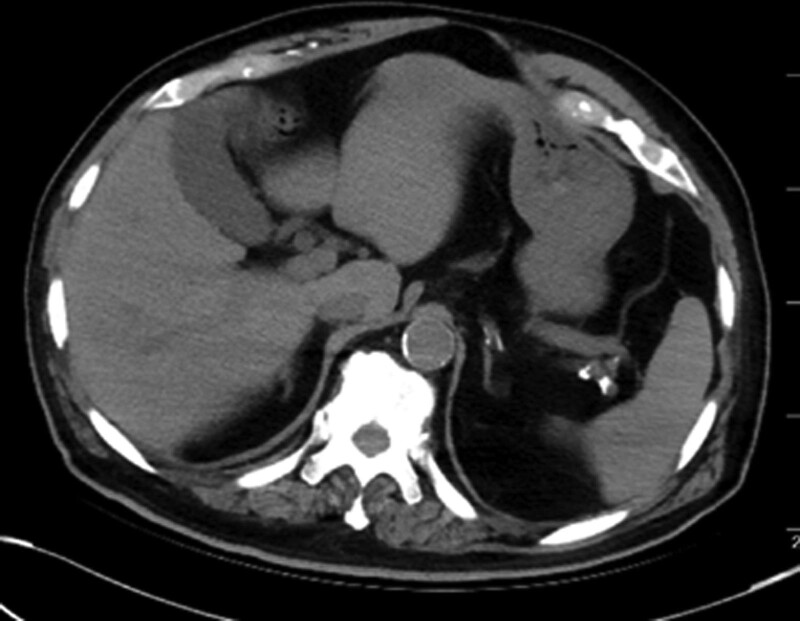
The contrast delayed in the right kidney and gallbladder has been excreted.

After treatment, the patient’s abdominal pain symptoms gradually improved, and all indicators including hemodynamics, renal function, and urine volume were normal, also the hemoglobin gradually raised up to normal levels. The abdominal CT scan showed the contrast medium disappeared and the kidney hemorrhage absorbed partly before discharge (Figs. 3 and 4). Three-month follow-up showed the patient was in good condition and presented with normal renal function.

## 3. Discussion

Local complications, such as puncture local hematoma, arterial dissection, arteriovenous fistula, and pseudoaneurysm, are common complications of cerebral angiography. And such hematomas after percutaneous angiography often occur in the groin, thigh, retroperitoneal, intraperitoneal, or abdominal wall. While hematomas in distant parts such as renal hematoma after percutaneous angiography are very rare.^[1,2]^ However, it is important to emphasize that active renal hematoma is a life-threatening complication after endovascular procedures. Therefore, the prevention of renal hematoma must be attached great importance during the intravascular treatment process, and the timely diagnosis and effective treatment should be provided once it occurs.

Renal hematomas could happen spontaneously or iatrogenically. The causes of spontaneous bleeding include neoplasm, bleeding tendency, vasculitis, and infection , while renal biopsy, extracorporeal lithotripsy, and anticoagulants belong to the iatrogenic causes.^[3]^ It was reported that the incidence of iatrogenic injuries which are usually caused by wires, catheters, or sheaths insertion after percutaneous renal artery revascularization had been 6.5% to 22.8%.^[1]^ The perforation and rupture of small branch vessels are usually caused by floppy-tipped guidewires, while stable wire position and J-shaped guide-wire could avoid these vascular injury.^[1]^ Therefore, careful manipulation of guide wires under full fluoroscopic navigation is crucial to prevention of renal hematoma during endovascular procedures.

The right renal hematoma in this case is considered to be related to the guide wire mistakenly entering the branch of the right renal artery. The patient did not have timely diagnosis as no obvious discomfort during the operation, and the postoperative abdominal pain was not typical. Therefore, the patients are recommended to be sent to the observation room for intensive-care at early postoperative period. Renal hematoma should be alerted if the patient complains of acute flank pain, especially who presents with progressively decrease of hemoglobin, hypotension, and tachycardia during or after the operative period. Computer tomography angiography has been regarded as an excellent diagnostic modality for hemorrhage with high sensitivity,^[4]^; however, it could delay an early effective treatment for patients with active bleeding and unstable vital signs. While bedside ultrasonography can be convenient and fast to detect renal hematoma, especially for patients who require expeditious intervention.^[5]^ We emphasize the importance of abdominal pain for unknown cause after interventional procedure, and the timely examination of abdominal ultrasound or CT for early diagnosis and treatment, in order to avoid catastrophic events.

Regarding the treatment of renal hemorrhage, most renal vascular injuries are self-healing. Conservative treatment is recommended for patients who are hemodynamically stable with no evidence of ongoing hemorrhage. It appears that there is no specialty for conservative treatment compared with renal artery embolization and surgical intervention. However, conservative treatments which development by multi-disciplinary collaboration (neurology, interventional surgery, urology, nephrology, and imaging department) including: hydration, diuretic therapy, anti-infection, analgesic drug combination, as well as monitoring renal function, hemoglobin, and hemodynamics, could prevent most kidney hemorrhage from surgical intervention and catastrophic events. This case highlights the importance of conservative treatment as foundational and critical therapy for kidney hemorrhage complicated by interventional therapy, which should not be overlooked.

Meanwhile, urgent intervention is indicated for patients with massive bleeding or progressively deterioration of the renal function. In such severe cases, intravenous bolus administration and blood transfusion could be effective to maintain vital signs stable for patients with systolic blood pressure rapidly decreased and the hemoglobin value sharply dropped. Compared with open surgery, renal angiography and subsequent selective embolization of bleeding vessels have been established as a preferred treatment for renal hemorrhage.^[6–8]^

Animal experiments show that iodixanol is mainly excreted by glomerular filtration after entering the human body, but about 7% part is excreted by bile and excreted from feces within 24 hours after injection.^[9]^ In our case, the contrast retention in the right kidney and gallbladder 24 hours after the operation might be due to the damage of the right kidney, while there were no significant abnormalities in renal function owing to the compensatory of the left kidney, and he maintained normal urine output and renal function. However, hemodialysis treatment may be needed for some patients with severe renal dysfunction due to renal hemorrhage and contrast injury.

## 4. Conclusion

The renal hematoma, commonly due to iatrogenic injury, is rare but life-threatening complication after endovascular procedures.^[10]^ Careful guide-wire passage under full visual inspection is important to avoid inadvertent vascular injury during percutaneous angiography. Meanwhile close surveillance and the awareness of this rare complication is crucial for the survival.

## Author contributions

YL, XZ, QY, GL, TL, and XZ contributed to the search and assessment of the available literature. YL and MZ wrote the manuscript. YZ and MZ helped revise the text to the final form. All authors contributed to the article and approved the submitted version.

**Conceptualization:** Yang Li, Xin Zhao, Ying Zhang, Qian Yang, Guoxing Liu, Tao Liu, Xuekai Zhang, Ming Zhou.

**Data curation:** Yang Li, Guoxing Liu, Ming Zhou.

**Formal analysis:** Yang Li.

**Funding acquisition:** Yang Li.

**Resources:** Tao Liu.

**Writing – original draft:** Yang Li, Ying Zhang, Xuekai Zhang, Ming Zhou.

**Writing – review & editing:** Yang Li, Xuekai Zhang, Ming Zhou.
